# Presbyopia correction with a new Extended Depth of Focus Intraocular Lens

**DOI:** 10.22336/rjo.2022.46

**Published:** 2022

**Authors:** Rozaliya Hristova, Galateya Tsvetkova, Denitsa Cholakova, Gergana Ivanova, Vassil Haykin

**Affiliations:** * Department of Ophthalmology, Medical University of Sofia, Bulgaria; ** Department of Ophthalmology, Alexandrovska University Hospital, Sofia, Bulgaria

**Keywords:** EDoF lens, presbyopia, cataract surgery

## Abstract

Extended depth of focus intraocular lenses (EDoF IOLs) offer an expanded number of modalities for simultaneous cataract and presbyopia treatment. The objective of the current study was to assess clinical outcomes with a new mono-EDoF intraocular lens and to analyze the effect of different parameters on postoperative results. The inclusion criteria were defined as uneventful cataract surgery, no history of concomitant ocular disease, implantation of ZOE Primus-HD lens. Parameters from IOL Master 500 were analyzed. The main outcome measures were postoperative uncorrected distance (UDVA) and intermediate (UIVA) visual acuity. The study included 39 eyes of 37 patients (15 males and 22 females) with a mean age of 73.59±7.71. Postoperatively, the UDVA improved to 0.84±0.16 (*p*<0.001) and UIVA was 0.86±0.14.

There was no correlation between K1, K2 and IOL power with both postoperative UDVA and IDVA. Moreover, there was no statistically significant difference between UDVA and UIVA between patients with mean K value over or under 44.0D (*p*=0.204 and *p*=0.817, respectively). The results of a multinomial logistic regression analysis for the predictive value of the factors K1, K2 and IOL power demonstrated no statistical significance, except for UIVA with a significant influence of IOL power (*p*=0.024) in patients with less than 0.9 Snellen visual acuity. The implantation of the new mono-EDoF ZOE Primus-HD lens led to improvement in both UDVA and UIVA. Patients with keratometry values less than 44.0D could still benefit from the mono-EDoF lenses. Further studies including wavefront aberrometry are needed to study the interaction between corneal aberrations and EDoF IOLs.

**Abbreviations:** IOL = Intraocular lens, UDVA = Uncorrected distance visual acuity, UIVA = Uncorrected intermediate visual acuity, D = Diopters, EDoF = Enhanced Depth of Focus, MF IOL = Multifocal intraocular lens, AUC = area under the curve

## Introduction

One of the most important factors in achieving the greatest possible postoperative refractive satisfaction in patients with implanted intraocular lenses is the capability of the IOL for extended range of vision with a far-point performance and minimization of photic phenomena [**1**]. 

EDoF lenses create a single elongated focal point to increase the depth of focus in contrast to monofocal (where light is fixed at one point) or multifocal IOL (two or three points) [**2**]. There are two known types of EDOF intraocular lenses (IOL): pure EDOF IOLs (equally refractive or diffractive) and hybrid MF-EDOF IOLs (refractive, diffractive and combined) [**3**]. What characterizes the pure EDOF IOLs is the spherical aberration-based optics or achieving the pinhole effect. They have a continuous optical profile, without changing the optical transition. Nevertheless, these lenses have no multifocality [**4**]. 

The spherical aberration is connected to the difference in the focal length between the central and peripheral rays passing through the lens. The corneal spherical aberration can be neutralized to a certain extent by choosing an IOL with negative spherical aberration [**5**]. The correction of spherical aberrations results in a better focus of light and better vision at a certain distance. Although higher order aberrations reduce the quality of vision in most cases, the presence of some of them (spherical aberration, coma, secondary astigmatism) improves the depth of focus. This is the main mechanism that allows the increase of the EDoF lenses depth of the focus.

The chromatic aberration is a consequence of the difference in the focal distance of rays with different colors. The cornea induces chromatic aberration as the blue light is subject to a larger diffraction as the red. Dispersion of the optical material, indexed with the Abbe number, indicates the extent to which the refractometric index is altered when the wavelength change is modified. The bigger the dispersion, the lower the Abbe number. Of relevance is also the optical design - the refractive optics retains the chromatic aberration of the cornea, which means that the resulting chromatic aberration will increase by increasing the dispersion of the different wavelengths [**6**]. The diffractive lenses can reverse the chromatic aberration. The achromatization does not lead to an increase in the depth of the field, but an improvement in the contrast sensitivity.

The ZOE Primus-HD is a monofocal intraocular lens that has an extended depth of focus (EDoF). The ZOE EDoF lens is a hydrophobic-acrylic lens with the following characteristics: overall diameter of 13 mm and 6 mm optic diameter, refractive index of 1.47, Abbe number of 57, 360° square edge, and 1.5° loop angle. Its aspheric design lowers the primary spherical aberrations of the cornea by -0.20 μm at 6 mm. The mono-EDoF effect relies on the high-order aspheric surface of the lens. The increased spherical aberration in the IOL center gradually decreases towards the periphery. The photic phenomena are minimized by the smooth and continuous higher-order aspheric surface.

Our choice of lens was supported by the lens’ expanded number of modalities for simultaneous cataract and presbyopia treatment.

The purpose of this study was to assess clinical outcomes with a new mono-EDoF intraocular lens and to analyze the effect of different parameters on postoperative results.

## Methods

This was a prospective single-arm non-blinded study on the clinical results following implantation of a new EDOF lens conducted from May 2021 to March 2022. It was approved by the Ethical Committee of Medical University of Sofia. All the patients signed an informed consent prior to participation in the study. All procedures involving human participants were performed in accordance with the 1964 Helsinki declaration and its later amendments or comparable ethical standards.

All the patients underwent a complete ophthalmological examination, including visual acuity, tonometry, optical coherence tomography, gonioscopy and confrontational visual fields to exclude ocular comorbidity. Inclusion criteria were uneventful cataract surgery, implantation of EDOF IOL ZOE, cortical or nuclear cataract, no history of concomitant ocular disease including keratoconus, glaucoma, uveitis, trauma, macular degeneration, etc., no history of previous ocular surgery. Only patients without postoperative complications, including posterior capsular opacification for the duration of the study or postoperative fibrinous exudate, were included. All surgeries were performed by a single ophthalmologist with more than 10 years of experience. 

For IOL power calculations, IOL Master Model 500 (Carl Zeiss Meditec, Dublin, CA) was used. Only scans with OK marking and SD less than 0.02 were included. The power of the lens was selected based on the SRKT and Holladay 1 formulas.

The surgical procedure was a standard technique of phacoemulsification under topical anesthesia with proxymetacaine hydrochloride. Only patients with superiorly placed main incision were included. No pupil dilating devices were used. Viscoelastic devices were applied using the soft-shell technique. The lens was implanted in the capsular bag. At the end of the surgery, prophylactic antibiotic (cefuroxime 0.01%) was injected intracamerally and dexamethasone subconjunctivally. Postoperative regiment included topical antibiotic (Ofloxacin) and steroid drops (Dexamethasone) four times daily in the operated eye.

The main outcome measures were postoperative uncorrected distance (UDVA), as assessed with Snellen charts and intermediate (UIVA) visual acuity, as assessed with Leo Numbers intermediate visual acuity chart with decimal representation. Secondary analysis on the effect of K1, K2, mean K values and IOL power was done to assess factors influencing postoperative visual acuity. IBM SPSS Statistics for Windows, Version 26.0 (IBM Corp., Armonk, NY, USA) was used for statistical analysis.

## Results

The study included 39 eyes of 37 patients (15 males and 22 females). No sex-based or racial/ ethnic-based differences were present. The mean age was 73.59±7.71 years. Mean preoperative K1, K2 values and mean IOL power were 43.47±1.44 (40.78 to 46.42), 44.24±1.46 (41.74 to 47.38) and 20.82±2.87D (12.0-26.0D), respectively. Mean preoperative uncorrected Snellen distance visual acuity was 0.16±0.19. Postoperatively, the uncorrected distance visual acuity improved to 0.84±0.16 (*p*<0.001) and intermediate uncorrected visual acuity was 0.86±0.14. The correlation between postoperative UDVA and UIVA was statistically significant (*r*=0.628; *p*<0.0001).

Pearson analysis of correlation between K1, K2 and IOL power with both postoperative distance and intermediate visual acuity did not demonstrate significant results. However, the correlation between distance visual acuity and IOL power was almost significant at *p*=0.062 (**Table 1**).

**Table 1:** Correlation between keratometry values (K1, K2), IOL power and visual acuity

Parameter	UDVA	UIVA
Plus or minus 0.5	*p*=0.158	*p*=0.150
K1	*p*=0.117	*p*=0.225
K2	*p*=0.153	*p*=0.431
IOL diopters	*p*=0.062	*p*=0.224

We further analyzed the data by dividing the patients into two categories based on their mean keratometry values – below 44.0D (n=18) and over 44.0D (n=21). There was no statistically significant difference between distance and intermediate visual acuity between the two groups (*p*=0.204 and *p*=0.817, respectively) on Kruskal-Wallis nonparametric test.

The results of a multinomial logistic regression analysis were used to identify the predictive value of factors including K1, K2 and IOL power on the postoperative visual acuity. The results are illustrated in **Table 2**. There was no statistical significance, except for intermediate visual acuity with a significant influence of the power of the IOL (*p*=0.024) in patients with less than 0.9 Snellen visual acuity (**Table 3**).

**Table 2:** Multinomial logistic regression analysis on the effect of K1, K2 and IOL power on postoperative distance visual acuity. The reference category is Snellen VA>0.9

Parameter	Sig.	Exp (B)	95% Confidence Interval
K1	.912	1.078	0.28-4.1
K2	.732	1.267	0.33-4.91
IOL power	.257	.843	0.63-1.13

**Table 3:** Multinomial logistic regression analysis on the effect of K1, K2 and IOL power on postoperative intermediate visual acuity. The reference category is visual acuity >0.9

Parameter	Sig.	Exp (B)	95% Confidence Interval
K1	.401	1.947	0.41-9.22
K2	.769	.794	0.17-3.71
IOL power	.024	.641	0.44-0.94

The corresponding areas under the curve (AUC) for the K1, K2 and IOL power are demonstrated in **Table 4**. An additional analysis was performed in the two groups based on keratometry values – under or over 44.0D. The results did not show statistically significant roles of K1, K2 or IOL power, as well as between patients with mean keratometry values over and under 44.0D.

**Table 4:** ROC analysis on the effect of K1, K2 and IOL power on predicting the postoperative result regarding distance and intermediate VA. Additionally, the keratometry values were divided into two categories – over or under 44.0D

	DISTANCE VA			INTERMEDIATE VA		
Parameter	AUC	95% CI	p-value	AUC	95% CI	p-value
K1	0.237	0.00-0.53	0.374	0.197	0.00-0.45	0.307
K2	0.237	0.00-0.53	0.374	0.711	0.36-1.0	0.477
IOL power	0.764	0.57-0.96	0.133	0.722	0.51-0.94	0.206
K value (<44.0 or >44.0)	0.635	0.45-0.82	0.175	0.577	0.39-0.77	0.439

## Discussion

The evolution of EDoF lenses has been unravelling at a fast pace. The main factor for this development is patient satisfaction especially with intermediate visual acuity. While some of the diffractive multifocal and EDoF lenses could reduce contrast sensitivity and cause visual phenomena, mono-EDoF lenses have certain advantages in this aspect [**7**]. Patient selection is important so that all the pros of the lens can be utilized while minimizing the cons.

This prospective study aimed to assess the results of a new EDoF lens. The main outcome measure was uncorrected distance and intermediate visual acuity. Our findings confirmed the results of previous studies on different EDoF lenses [**4**]. Although patients with intraocular complications such as poor dilation, intraoperative floppy iris syndrome, zonular weakness, etc., were excluded from the current study, they could still benefit from a mono-EDoF lens [**8**]. We found statistically significant improvement in visual acuity in all patients.

This was an interesting result given that keratometry values ranged from 40.87 to 47.38. Even though the manufacturer recommends using the lens primarily in patients with keratometry values over 44.0D, we did not find a statistically significant predictor role for this parameter. However, increasing IOL power, the chances of having UIVA less than 0.9 increased. This could be interpreted as an indication that choosing a lens with lower power results in better UIVA without compromising UDVA [**9**].

Pupil diameter is known to affect depth of field. The amount of light entering the eye is proportional to the pupil area. Therefore, the pupil size could have influenced our results and explains the optimal results in patients with lower keratometry values. However, we did not include pupil size since the IOL Master does not take it into account when calculating the IOL power.

As in other studies, we observed that corneal spherical aberration varies greatly among individuals [**10**]. In both cases – refraction of the outer paraxial rays by far greater amount than central rays (positive spherical aberration) and refraction of the outer paraxial rays by a lesser amount than central rays (negative spherical aberration) – the end effect causes a longitudinal spread of focus. The elimination of the caused “blur” with pure EDoF lenses elongating the focus or using the pinhole effect was registered, however, lack of multifocality at the cost of degraded quality of vision could have been present. Hopefully, further studies will prove EDoF lenses successfully compensate for the spherical aberrations of the cornea in intermediate vision, and perform with minimal photic effects at distance.

Apart from keratometry values, other considerations for preoperative patient evaluation are anterior chamber depth, iridocorneal complex (e.g. iris plateau [**11**]), concomitant ocular diseases, such as age-related macular degeneration, glaucoma, diabetic retinopathy, etc. Although we included only patients without history of ocular disease, different anatomical variations could have influenced the effective lens position – the most unpredictable variable in IOL calculations.

The limitation of our study was the lack of corneal topography, wavefront aberrometry and contrast sensitivity testing. Corneal topography and wavefront aberrometry would have given more information on corneal aberrations, however, they are usually implemented in more complex eyes, e.g. post-LASIK, significant astigmatism, etc. Contrast sensitivity is an important issue in multifocal lenses, but we did not conduct it because the IOL used was pure EDoF and the main outcome measures were distance and near visual acuity. The non-randomized nature of the study and the lack of comparison with other types of IOLs could also be viewed as a disadvantage.

## Conclusion

The implantation of the new mono-EDoF ZOE Primus-HD lens leads to improvement in both uncorrected distance and intermediate visual acuity. Patients with keratometry values less than 44.0D could still benefit from mono-EDoF lenses. Further studies, including wavefront aberrometry, are needed to study the interaction between corneal aberrations and EDoF IOLs.

**Conflict of Interest statement**

All authors declare that there is no conflict of interest regarding the publication of this paper.

**Informed Consent and Human and Animal Rights statement**

Informed consent has been obtained from all individuals included in this study.

**Authorization for the use of human subjects**

Ethical approval: The research related to human use complies with all the relevant national regulations, institutional policies, is in accordance with the tenets of the Helsinki Declaration, and has been approved by the Ethical Committee of the Medical University of Sofia, Bulgaria.

**Acknowledgements**

The authors acknowledge the contribution of all staff members of Department of Ophthalmology, Alexandrovska University Hospital.

**Sources of Funding**

This work did not receive any funding.

**Disclosures**

None.
